# Multistage Fragmentation of Ion Trap Mass Spectrometry System and Pseudo-MS^3^ of Triple Quadrupole Mass Spectrometry Characterize Certain (*E*)-3-(Dimethylamino)-1-arylprop-2-en-1-ones: A Comparative Study

**DOI:** 10.1155/2014/702819

**Published:** 2014-02-19

**Authors:** Ali S. Abdelhameed, Adnan A. Kadi, Hatem A. Abdel-Aziz, Rihab F. Angawi, Mohamed W. Attwa, Khalid A. Al-Rashood

**Affiliations:** ^1^Department of Pharmaceutical Chemistry, College of Pharmacy, King Saud University, P.O. Box 2457, Riyadh 11451, Saudi Arabia; ^2^Department of Applied Organic Chemistry, National Research Center, Dokki, Cairo 12622, Egypt; ^3^Department of Chemistry, College of Science, King Abdulaziz University, P.O. Box 54881, Jeddah 21589, Saudi Arabia

## Abstract

A new approach was recently introduced to improve the structure elucidation power of tandem mass spectrometry simulating the MS^3^ of ion trap mass spectrometry system overcoming the different drawbacks of the latter. The fact that collision induced dissociation in the triple quadrupole mass spectrometer system provides richer fragment ions compared to those achieved in the ion trap mass spectrometer system utilizing resonance excitation. Moreover, extracting comprehensive spectra in the ion trap needs multistage fragmentation, whereas similar fragment ions may be acquired from one stage product ion scan using the triple quadrupole mass spectrometer. The new strategy was proven to enhance the qualitative performance of tandem mass spectrometry for structural elucidation of different chemical entities. In the current study we are endeavoring to prove our hypothesis of the efficiency of the new pseudo-MS^3^ technique via its comparison with the MS^3^ mode of ion trap mass spectrometry system. Ten pharmacologically and synthetically important (*E*)-3-(dimethylamino)-1-arylprop-2-en-1-ones (enaminones **4a–j**) were chosen as model compounds for this study. This strategy permitted rigorous identification of all fragment ions using triple quadrupole mass spectrometer with sufficient specificity. It can be used to elucidate structures of different unknown components. The data presented in this paper provide clear evidence that our new pseudo-MS^3^ may simulate the MS^3^ of ion trap spectrometry system.

## 1. Introduction

Mass spectrometry is a very efficient tool for the analysis of drugs, proteins, peptides, and so forth. Triple quadrupole mass spectrometer system (QqQ) has always been noted for the use in quantitative analysis of the various compounds, while ion trap mass spectrometry system (IT) is a well known tool for qualitative analysis. Our previously published work [1] about in-source fragmentation and what was referred to as pseudo-MS^3^ has demonstrated an optimistic way to use the triple quadrupole mass spectrometer system (QqQ) for qualitative analysis with relatively equal capabilities of ion trap system. Although this in-source fragmentation technique can produce various mass fragments derived from the different compounds in the ion source—which confuses the determination of fragment ions derived from the investigated compounds—, it has been widely used by several research groups [2–11]. However, the use of collision induced dissociation (CID) in the QqQ generates rich fragment ions in a single-stage product ion (MS^2^) scan, which differs from the less sensitive and time consuming multistage fragmentation (MS^*n*^) in case of CID via in-trap resonance excitation. Additionally, ion trap cannot attain the parent ion and the lowest *m*/*z *daughter ion stable at the same time in the trap (low mass cut-off) in MS^2^ scans. [12, 13]. To prove the capability of our pseudo-MS^3^ hypothesis, a comparison with the real MS^3^ in the ion trap is of great importance. In the present study, we use pseudo-MS^3^ approach using electrospray ionization tandem mass spectrometry (ESI-MS/MS) and MS^3^ of IT to obtain structural information of certain novel enaminones. The use of this pseudo-MS^3^ approach was primarily intended to raise the qualitative efficiency of QqQ bringing it to similar qualitative capability of IT with none of the previously mentioned drawbacks of IT.

Ten substituted (*E*)-3-(dimethylamino)-1-arylprop-2-en-1-ones (enaminones) were selected for this comparative study. Enaminones are important synthons for the constructions of a wide variety of biologically active compounds and a huge number of reports were published that deal with the chemistry of enaminones due to their role in the synthetic chemistry [14–18]. Thus, there is necessity to improve the known analyses methods for this class of compounds or develop new methods. In continuation for our previous work [19–24] in the chemistry of the title compounds, certain enaminones **4a–j **were synthesized and used as model compounds for this current study.

## 2. Materials and Methods

Unless otherwise indicated, all chemicals were purchased from Sigma (St. Louis, MO).

### 2.1. Chemistry

**Figure 1:**
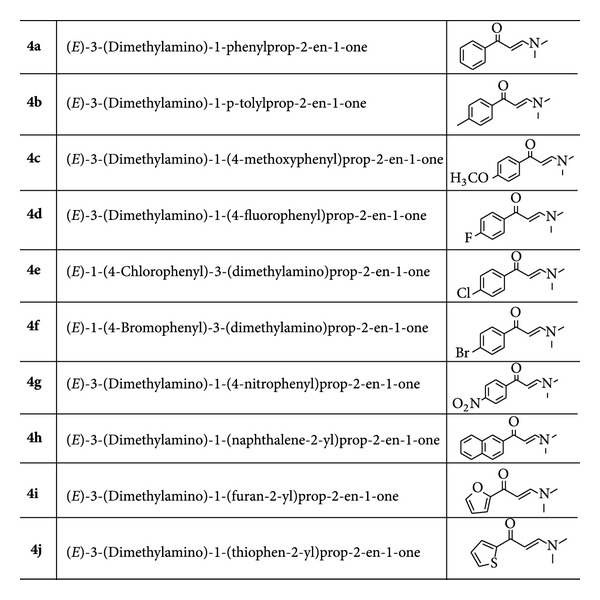
The structures of the selected enaminones **4a–j**.

(*E*)-3-(dimethylamino)-1-arylprop-2-en-1-ones **4a–j**  (Figure 1) were synthesized according to the reported method by Saleh et al. [23] and crystallized from EtOH to enhance sample purity for analysis.

### 2.2. Mass Spectrometry

#### 2.2.1. Reagents and Solvents

HPLC water was purified using cartridge system (Milford, Bedford, USA) (Ultrapure water of 18 *μ*Ω was obtained from Milli-Q plus purification system (Millipore, Bedford, MA, USA). Acetonitrile (ACN) HPLC grade was purchased from BDH laboratory supplies (Poole, UK).

#### 2.2.2. Triple Quadrupole Mass Spectrometry (QqQ)

An Agilent 6410 triple quadrupole mass spectrometer (Agilent technologies, USA) equipped with an electrospray ionization interface (ESI) coupled to an Agilent 1200 HPLC (Agilent Technologies, USA) was used. Agilent 1200 series system consists of G1311A binary pump, G1322A degasser, G1367B HIP-ALS autosampler, and G1316A thermostatted column compartment. A connector is used instead of the column to allow direct injection of samples. Mobile phase was composed of two solvents: A is HPLC grade water, and B is acetonitrile mixed in the ratio 1 : 1. Compounds were prepared by weighing the solid substances to 1 mg mL^−1^ in ACN. Test solutions for MS were prepared by diluting the stock solutions with mobile phase. Flow rate was 0.4 mL min^−1^ and run time was 3 minutes. 10 *μ*L of each sample was injected into the LC-MS/MS. MS parameters were optimized for each compound by varying fragmentor voltage of the ion source for scan mode and collision energy for product ion mode. For optimization of the ionization conditions and of fragment ion spectra, analytes concentration of 10 *μ*g mL^−1^ was employed. For screening of mass signals of the different compounds and to search for parent ions for MS/MS experiments, MS2 scans were performed in the mass range of *m/z* 100–600. Because of the flow rate dependency of the ESI process, ion source specific parameters were readjusted. The ESI was operated in positive mode. The source temperature was set to 350°C and ion spray voltage was 4.5 kV.

To achieve high selectivity using in-source fragmentation, a preliminary MS^2^ scan was carried out prior to the in-source fragmentation to investigate each compound's related fragment ions. The fragmentor voltage was optimized to produce sufficient in-source fragmentation; values of 100, 120, 140, 160, 180, and 200 V were tested to obtain the fragments of each compound in the scan spectra. The optimum fragmentor voltage to generate in-source fragments was 180 V. Furthermore, the collision energy used for product ion (MS^2^) analysis was also optimized by varying collision energy values (4, 6, 8, 10, 12, 14, 16, 18, and 20 eV) and was set to 16 eV to attain the fragment ions.

#### 2.2.3. Ion Trap Mass Spectrometry (IT)

An Agilent 6320 Ion trap mass spectrometer (Agilent technologies, USA) equipped with an electrospray ionization interface (ESI) was used. A connector is used instead of the column to allow direct injection of samples. Mobile phase was composed of a mixture of solvents A and B (1 : 1), where A is HPLC grade water and B is acetonitrile. Compounds were prepared by weighing the solid substances to 1 mg·mL^−1^ in mobile phase. Test solutions were prepared by diluting the stock solutions to 10–30 *μ*gmL^−1^—depending on the ions intensities—with mobile phase. Flow rate was 0.2 mL min^−1^ and run time was 4 minutes. MS parameters were optimized for each compound. The scan was ultrascan mode. For screening of mass signals of the different compounds and to search for parent ions for MS/MS experiments, MS2 scans were performed in the mass range of *m/z* 50–400. The ESI was operated in positive mode. The source temperature was set to 350°C nebulizer gas pressure of 55.00 psi, dry gas flow rate of 12.00 L min^−1^.

## 3. Results and Discussion

### 3.1. Chemistry

**Scheme 1:**
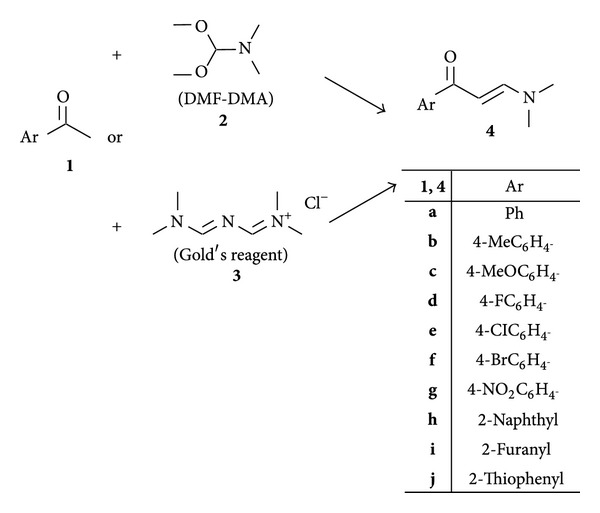
Structure of compounds **1–4a–j**.

Several methods for the preparation of enaminones have been reported [24]. Enaminones **4a–j** were efficiently prepared by the reaction of ketone **1a–j** with dimethylformamide-dimethylacetal (DMF*-*DMA) (**2**) or with [3-(dimethylamino)-2-azaprop-2-en-1-ylidene] dimethylammonium chloride (Gold's reagent) (Scheme 1) (**3**).

### 3.2. Mass Spectrometry

#### 3.2.1. Triple Quadrupole Mass Spectrometer (QqQ)

**Figure 2:**
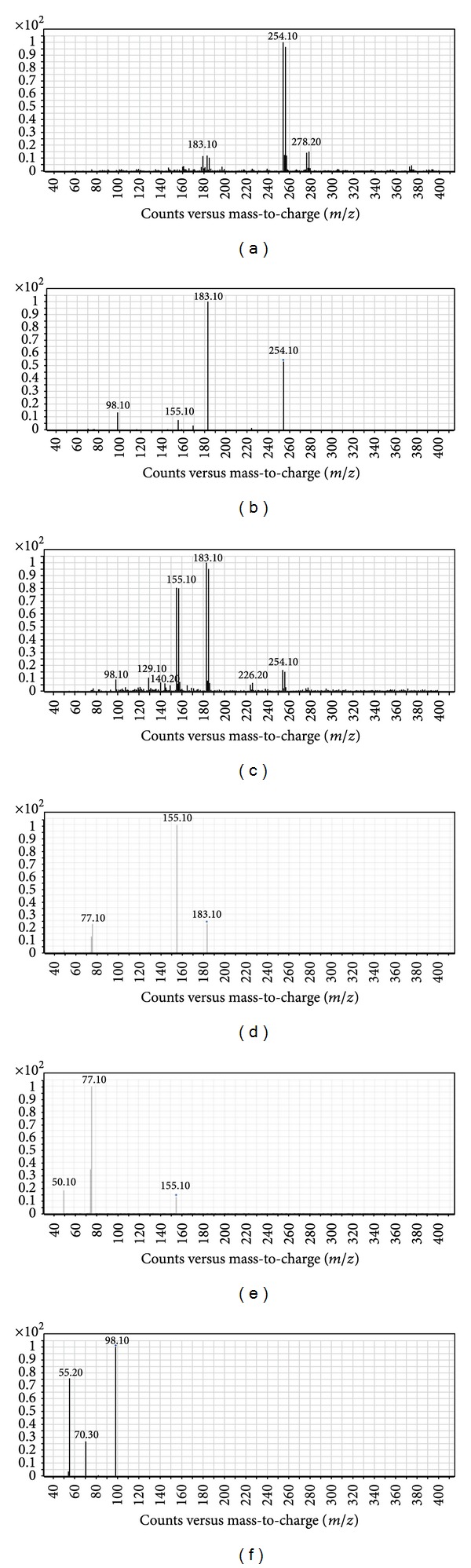
(a) ESI mass spectrum of compound **4f** [M + H]^+^ ion (*m/z* 254.10). (b) MS^2^ spectrum of *m/z *254.10. (c) In-source fragmentation of compound **4f**. (d) MS^2^ spectrum of *m/z* 183.10. (e) MS^2^ spectrum of *m/z *155.10. (f) MS^2^ spectrum of* m/z* 98.10.

**Figure 3:**
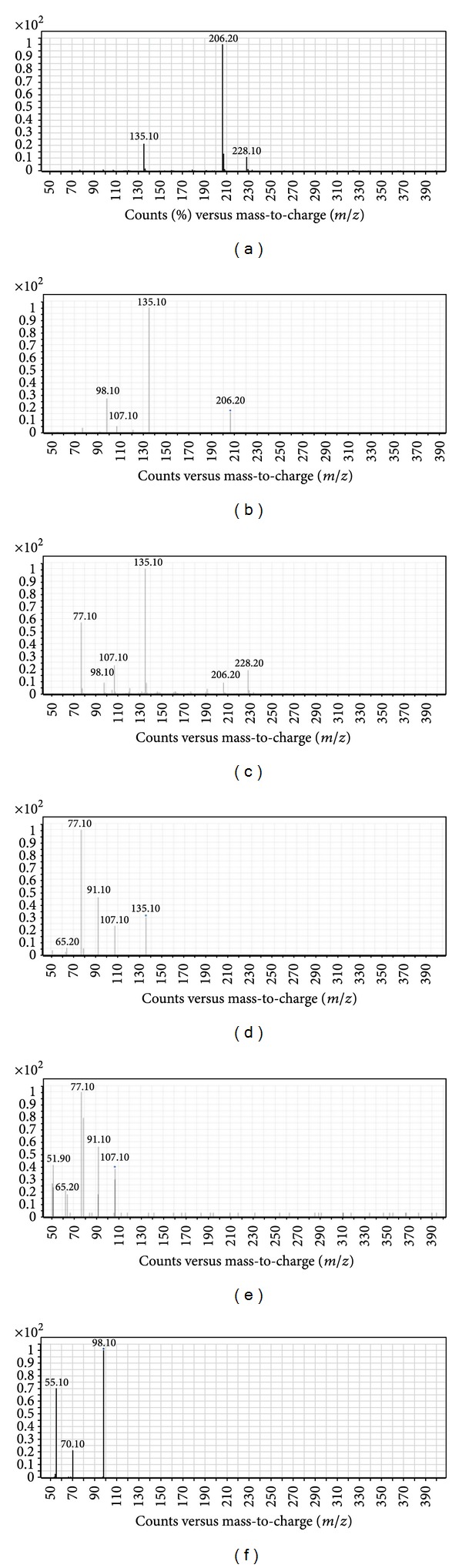
(a) ESI mass spectrum of compound **4c** [M + H]^+^ ion (*m/z* 206.20). (b) MS^2^ spectrum of *m/z *206.20. (c) In-source fragmentation of compound **4c**. (d) MS^2^ spectrum of *m/z* 135.10. (e) MS^2^ spectrum of *m/z *107.10. (f) MS^2^ spectrum of* m/z* 98.10.

For pseudo-MS^3^, an initial MS2 scan followed by a product ion scan of each compound was performed to distinguish the parent ion peaks as well as the fragment ions of compounds **4a–j**. The data obtained played a guidance role prior to the pseudo-MS^3^ process for the same compounds. The highly sensitive product ion spectra of compounds **4a–j** obtained from a single-stage MS^2^ scan with abundant product ions and no low mass cut-off are represented in Figures 2 and 3. The in-source fragmentation step revealed various fragments including these daughter ion peaks produced by MS/MS scans, which in turn were used as precursor ions for pseudo-MS^3^ step.

**Scheme 2:**
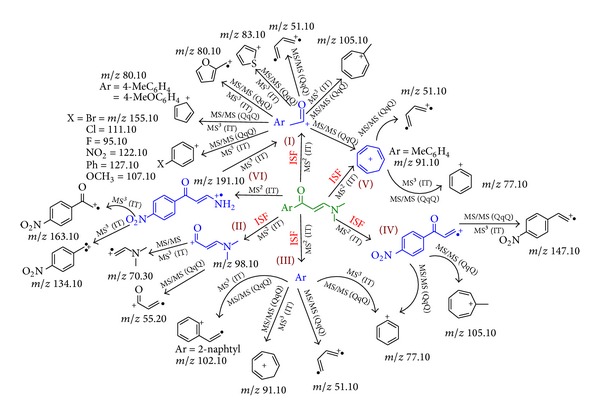
Proposed fragmentation pattern of compounds **4a–j** conducted with QqQ and IT.

The fragmentation pattern of compounds **4a–j **was investigated using triple quadrupole mass spectrometry and ion trap mass spectrometry system (Scheme 2). In triple quadrupole pseudo-MS^3^, a pattern of five major fragments (I–V) was observed following in-source fragmentation (ISF) for all compounds **4a–j** regardless of the different aryl groups of the main nucleus. Regardless of the different substituents of compounds **4a–j**,one fragment (I) was commonly observed in their spectra with [M + H]^+^ at *m/z* (105.10, 119.10, 135.10, 123.10, 139.20, 183.10, 150.30, 155.10, 95.10 and 111.10 for compounds **4a–j **resp.). These [M + H]^+^ values suggested that this fragment is characterized by the removal of N-dimethylprop-1-en-1-amine group. Upon exposure to further MS/MS fragmentation fragment (I) showed defined pattern of freeing the aryl group to produce five different fragments, one at [M + H]^+^ at *m/z* (107.10, 95.10, 111.10, 155.10, 122.10, 127.10, 80.10 and 83.10 for **4c**,** d**,** e**,** f**,** g**,** h**,** I**,and** j**, resp.). Other two fragments of compound (I) were suggested to undergo ring rearrangement to yield a substituted tropilium ion [M + H]^+^ at *m/z* 105.10 in case of **4a** and a typical tropilium ion [M + H]^+^ at *m/z* 91.10 at in case of **4b**. Additionally, fragment (I) produced a suggested buta-1,3-diene fragment [M + H]^+^ at *m/z* 51.10 for compounds **4a–g** and cyclopenta-2,4-dien-ilium ion [M + H]^+^ at *m/z* 65.10 for **4b** and **4c**. Fragment (II) was observed in the spectra of ISF in all compounds **4a–j** [M + H]^+^ at *m/z* 98.10 assuming the loss of the aryl group. Following further MS/MS fragmentation of this daughter ion peak, it produces consistent two fragments [M + H]^+^ at *m/z* 70.30 and 55.20, that perhaps was characterized by the removal of a acetaldehyde and dimethylamine moieties, respectively. Moreover, fragment (III) was also observed in all mass spectra of ISF except compounds **4i** and** 4j** [M + H]^+^ at *m/z* (77.10, 91.10, 107.10, 95.10, 111.10, 155.10, 122.10, and 127.10 for **4a–h** resp.), that suggested that this fragment is the aryl group of these compounds. Post-fragmentation spectra of this daughter ion peak revealed three common fragments [M + H]^+^ at *m/z* 77.10, 91.10 and 51.10 assuming a benzenium ion, tropilium ion, and cyclopenta-2,4-dien-1-ylium ion, respectively. A fourth fragment observed after MS/MS fragmentation of fragment (III) in case of compound **4h** was [M + H]^+^ at *m/z* 102.10 suggesting a styrene moiety. Another fragment of ISF (IV) [M + H]^+^ at *m/z* 175.10 appeared only in case of compound **4g** suggesting the loss of a trimethylamine moiety. This fragment (IV) via product ion yields [M + H]^+^ at *m/z* 77.10, 105.10, and 147.10 suggesting a benzenium ion, tropilium ion, and nitrovinylbenzene moieties, respectively Fragment (V) of ISF was a typical tropilium ion [M + H]^+^ at *m/z* 91.10 observed only in case of compound **4b**. All ion peaks together with their corresponding proposed structures obtained from ISF and MS^2^ scans for compounds **4a–j** are shown in Scheme 2 and are also summarized in Table 1.

#### 3.2.2. QqQ versus IT

**Figure 4:**
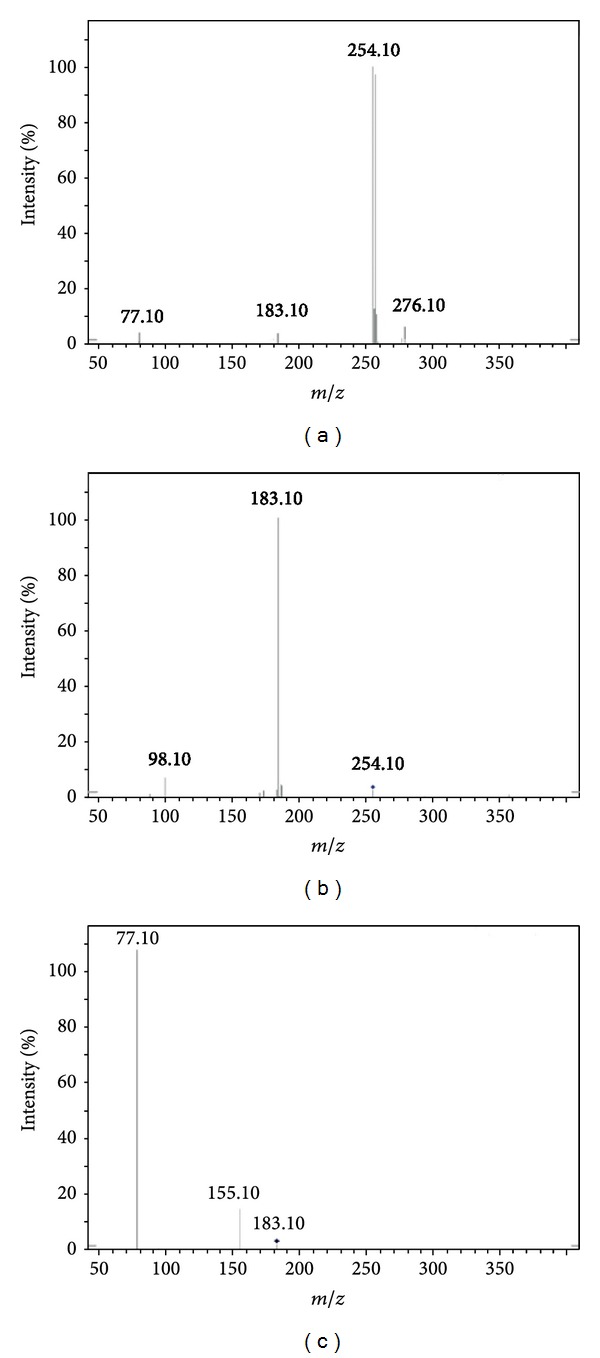
(a) Ion trap MS/MS mass spectrum of compound **4f** [M + H]^+^ ion (*m/z* 254.10). (b) MS^2^ spectrum of *m/z* 254.10. (c) MS^3^ spectrum of 183.10.

**Figure 5:**
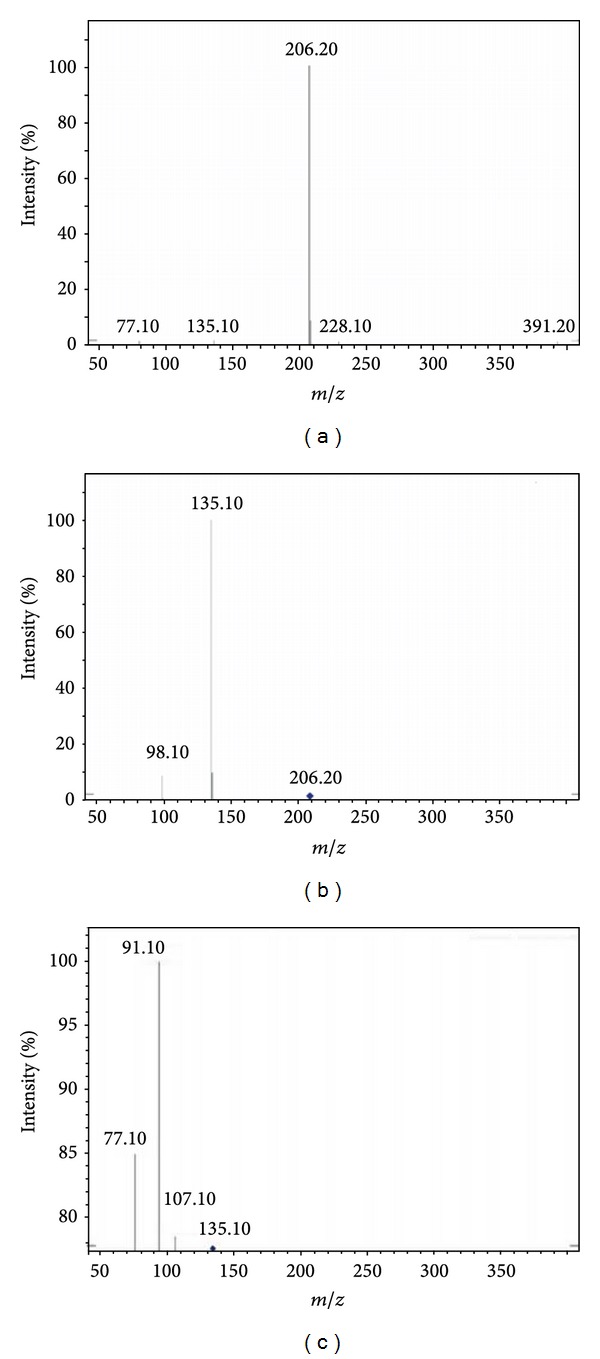
(a) Ion trap MS/MS mass spectrum of compound **4c** [M + H]^+^ ion (*m/z* 206.20). (b) MS^2^ spectrum of *m/z* 206.20. (c) MS^3^ spectrum of 135.10.

On the other hand, in the analysis using MS^3^ of IT, compounds were directly subjected to MS2 scan followed by product ion scans of the target precursor ion and fragmentation of resulting daughter ion peaks at the MS^3^ scan mode. Fragmentation pathway of the investigated compounds **4a–j** was studied thoroughly using IT. Fewer fragments were observed in IT spectra of the compounds than those obtained via QqQ. Additionally, the low mass cut-off effect of IT was remarkably clear when performing the fragmentation studies. Only one fragment (VI) was observed in IT and not in QqQ in the MS/MS which was further fragmented by MS^3^ to yield [M + H]^+^ at *m/z* 163.10, 150.30 and 134.10 that assumed loss of trimethylamine, N-dimethylprop-1-en-1-amine, and (dimethylamino-)acrylaldehyde moieties, respectively. For comparison purposes, representative spectra resulted from different stages of ion trap analysis are shown in Figures 4 and 5. Fragmentation ion peaks of ion tarp are also summarized in Table 1.

## 4. Conclusions

This study elaborated the fragmentation mechanism series of enaminones **4a–j** and compared their MS spectrum using both pseudo-MS^3^ of QqQ and MS^3^ of IT methods. More diverse fragmentation data was obtained using the pseudo-MS^3^ for all compounds. However, in IT fewer fragments were obtained and low mass cut-off was shown to be of great influence to the spectra observed, whereas, in QqQ, the product ion spectra of compounds **4a–j** were acquired from one stage MS^2^ scan with rich product ions and no low mass cut-off. Both fragmentation mechanisms have some advantages and disadvantages. The multistage fragmentation of IT (MS^*n*^) is more efficient for more complex compounds as one can utilize the various stages to further elucidate the unknown chemical structures. However, the pseudo-MS^3^ strategy is facile, adequately selective, sensitive, rapid, and rigorous for the identification of unknown compounds.

## Figures and Tables

**Table 1:** Multistage comparison MS data of compounds **4a–j** by pseudo-MS^3^of QqQ and/or MS^3^of IT.

MS Technique	**4a** (m/z 176.20)			**4b** (m/z 190.10)			**4c** (m/z 206.20)			**4d** (m/z 193.20)			**4e** (m/z 210.10)		
	m/z	QqQ	IT	m/z	QqQ	IT	m/z	QqQ	IT	m/z	QqQ	IT	*m*/*z*	QqQ	IT
ISF (QqQ)/MS^2^ (IT)	98.10	*✓*	*✓*	91.10	*✓*		98.10	*✓*		95.10	***✓***		98.10	***✓***	***✓***
	105.10	*✓*	*✓*	98.10	*✓*	*✓*	107.10	*✓*	***✓***	98.10	***✓***	***✓***	111.10	***✓***	
							119.10	*✓*		123.10	***✓***	***✓***	139.20	***✓***	***✓***
							135.10	*✓*	*✓*						

Pseudo-MS^3^ (QqQ)/MS^3^ (IT)	51.10	*✓*		51.10	*✓*		51.10	***✓***		51.10	***✓***		51.10	***✓***	
	55.20	*✓*		55.20	*✓*		55.20	***✓***		55.20	***✓***		55.20	***✓***	
	70.30	*✓*	*✓*	65.10	*✓*	***✓***	65.10	***✓***		70.30	***✓***		70.30	***✓***	
	77.10	*✓*	*✓*	70.30	*✓*	***✓***	70.30	***✓***		95.10	***✓***	***✓***	77.10	***✓***	***✓***
	105.10	*✓*	*✓*	77.10	*✓*	***✓***	77.10	***✓***	***✓***				111.10	***✓***	***✓***
				91.10	*✓*	***✓***	91.10	***✓***	***✓***						
							107.10	***✓***	***✓***						

MS Technique	**4f **(*m*/*z* 254.10)			**4g **(*m*/*z* 221.10)			**4h **(*m*/*z* 226.20)			**4i **(*m*/*z* 188.10)			**4j **(*m*/*z* 182.10)		
	*m*/*z*	QqQ	IT	*m*/*z*	QqQ	IT	*m*/*z*	QqQ	IT	*m*/*z*	QqQ	IT	*m*/*z*	QqQ	IT

ISF (QqQ)/MS^2^ (IT)	98.10	***✓***	***✓***	98.10	***✓***	***✓***	98.10	***✓***	***✓***	95.10	***✓***	***✓***	98.10	***✓***	***✓***
	155.10	***✓***		122.10	***✓***		127.10	***✓***	***✓***	98.10	***✓***	***✓***	111.10	***✓***	***✓***
	183.10	***✓***	***✓***	150.30	***✓***	***✓***	155.10	***✓***	***✓***						
				175.10	***✓***	***✓***									
				191.10	***✓***	***✓***									

Pseudo-MS^3^ (QqQ)/MS^3^ (IT)	51.10	***✓***		51.10	***✓***		55.20	***✓***		55.20	***✓***		55.20	***✓***	
	55.20	***✓***		55.20	***✓***		70.30	***✓***		70.30	***✓***	***✓***	70.30	***✓***	
	70.30	***✓***		70.30	***✓***		102.10	***✓***	***✓***	80.10	***✓***	***✓***	83.10	***✓***	***✓***
	77.10	***✓***	***✓***	105.10	***✓***	***✓***	127.10	***✓***	***✓***						
	155.10	***✓***	***✓***	122.10	***✓***	***✓***	147.10	***✓***	***✓***						
				134.10		***✓***									
				150.30	***✓***	***✓***									
				163.10		***✓***									
